# The cost-effectiveness of providing antenatal lifestyle advice for women who are overweight or obese: the LIMIT randomised trial

**DOI:** 10.1186/s40608-015-0046-4

**Published:** 2015-03-11

**Authors:** Jodie M Dodd, Sharmina Ahmed, Jonathan Karnon, Wendy Umberger, Andrea R Deussen, Thach Tran, Rosalie M Grivell, Caroline A Crowther, Deborah Turnbull, Andrew J McPhee, Gary Wittert, Julie A Owens, Jeffrey S Robinson, For the LIMIT Randomised Trial Group

**Affiliations:** School of Paediatrics and Reproductive Health, and The Robinson Research Institute, The University of Adelaide, Adelaide, Australia; Department of Perinatal Medicine, Women’s and Babies Division, The Women’s and Children’s Hospital, North Adelaide, Australia; Agricultural and Food Economics, Global Food Studies, Faculty of the Professions, The University of Adelaide, Adelaide, Australia; Women’s and Children’s Health Research Institute, North Adelaide, Australia; School of Population Health, The University of Adelaide, Adelaide, Australia; Liggins Institute, University of Auckland, Auckland, New Zealand; School of Psychology, The University of Adelaide, Adelaide, Australia; Department of Neonatal Medicine, Women’s and Babies Division, The Women’s and Children’s Hospital, North Adelaide, Australia; School of Medicine, The University of Adelaide, Adelaide, Australia

**Keywords:** Pregnancy, Overweight and obesity, Health economics evaluation, Cost effectiveness analysis, Randomised trial, dietary and lifestyle intervention

## Abstract

**Background:**

Overweight and obesity during pregnancy is common, although robust evidence about the economic implications of providing an antenatal dietary and lifestyle intervention for women who are overweight or obese is lacking.

We conducted a health economic evaluation in parallel with the LIMIT randomised trial. Women with a singleton pregnancy, between 10^+0^-20^+0^ weeks, and BMI ≥25 kg/m^2^ were randomised to Lifestyle Advice (a comprehensive antenatal dietary and lifestyle intervention) or Standard Care. The economic evaluation took the perspective of the health care system and its patients, and compared costs encountered from the additional use of resources from time of randomisation until six weeks postpartum. Increments in health outcomes for both the woman and infant were considered in the cost-effectiveness analysis. Mean costs and effects in the treatment groups allocated at randomisation were compared, and incremental cost effectiveness ratios (ICERs) and confidence intervals (95%) calculated. Bootstrapping was used to confirm the estimated confidence intervals, and to generate acceptability curves representing the probability of the intervention being cost-effective at alternative monetary equivalent values for the outcomes avoiding high infant birth weight, and respiratory distress syndrome. Analyses utilised intention to treat principles.

**Results:**

Overall, the increase in mean costs associated with providing the intervention was offset by savings associated with improved immediate neonatal outcomes, rendering the intervention cost neutral (Lifestyle Advice Group $11261.19±$14573.97 versus Standard Care Group $11306.70±$14562.02; p=0.094). Using a monetary value of $20,000 as a threshold value for avoiding an additional infant with birth weight above 4 kg, the probability that the antenatal intervention is cost-effective is 0.85, which increases to 0.95 when the threshold monetary value increases to $45,000.

**Conclusions:**

Providing an antenatal dietary and lifestyle intervention for pregnant women who are overweight or obese is not associated with increased costs or cost savings, but is associated with a high probability of cost effectiveness. Ongoing participant follow-up into childhood is required to determine the medium to long-term impact of the observed, short-term endpoints, to more accurately estimate the value of the intervention on risk of obesity, and associated costs and health outcomes.

**Trials registration:**

Australian and New Zealand Clinical Trials Registry (ACTRN12607000161426).

## Background

It has been estimated that in excess of 1.46 billion adults [1], and 170 million children [2] across the globe are obese. Obesity is a well-recognised contributor to the overall global burden of disease [3], associated with a range of adverse health outcomes including cardiovascular disease, type-2 diabetes and several cancers. When compared with individuals of normal body mass index, obesity has been conservatively estimated to increase the costs of health care by 30% for individuals who are obese, compared with those of normal body mass index (BMI) [4]. Furthermore, obesity is estimated to result in more than 36 million disability-adjusted life-years [5], and to contribute directly to in excess of 3.4 million deaths annually [6].

The financial costs of obesity are substantial, an Australian report estimating approximately $21 billion annually in 2005 [7], further increasing to more than $58 billion per annum in 2008 [8]. Using a broader concept of “cost” to include measures of collective wellbeing, it has been estimated that obesity accounts for $120 billion annually, or 8% of Australia’s national annual productivity [9]. Data from the United States indicate $147 billion dollars, or 10% of the country’s total health care expenditure was spent on treatment of obesity related complications in 2006 [10], with projections indicating a doubling of costs each decade, representing up to 18% of total health-care expenditure by 2030 [11].

Obesity represents a significant health burden for women and their infants during pregnancy and childbirth, with almost 50% of women having a BMI above 25 kg/m^2^ on entering pregnancy [12-14]. The effects of high maternal BMI on pregnancy and infant health have been well documented, the risks of adverse outcomes increasing with increasing maternal BMI [15-19]. While the economic implications associated with obesity have been relatively well described in non-pregnant settings [4,6-8], there is more limited information available relating to the healthcare costs associated with obesity during pregnancy. The limited available literature indicates maternal overweight and obesity to be associated with increased cost of healthcare, including prolonged hospitalisation and neonatal nursery care, when compared with women of normal BMI [20-24], estimated to be 23% higher among women who are overweight, and increasing further to 37% greater among women who are obese [22].

There is considerable research interest in the evaluation of antenatal interventions provided to women who are overweight or obese to limit gestational weight gain [25,26]. However, to our knowledge, there is little robust information available evaluating the economic implications of providing these interventions, either at an individual or population level. The aim of this pre-specified analysis was to evaluate the economic costs and consequences of providing an antenatal lifestyle intervention for women who are overweight or obese, conducted within the context of the LIMIT randomised trial [27].

## Methods

An economic evaluation was conducted in conjunction with the LIMIT randomised trial. The protocol [28] and clinical findings from the LIMIT trial have been published in detail previously [27,29,30]. Briefly, eligible women with a singleton pregnancy and BMI ≥25 kg/m^2^, who were between 10^+0^ and 20^+0^ weeks’ gestation, were recruited from the three major metropolitan maternity hospitals within Adelaide, South Australia. All women provided written informed consent, following approval from the ethics committee at each collaborating hospital including Women’s and Children’s Health Network HREC, Human Research Ethics Committee (TQEH/LMH/MH) and Southern Adelaide Clinical HREC. At the time of their first antenatal visit, all women had their height and weight measured, and BMI calculated. Women were then allocated a study number and randomised to receive either ‘Lifestyle Advice’ or ‘Standard Care’.

Women randomised to receive Lifestyle Advice participated in a comprehensive dietary and lifestyle intervention over the course of their pregnancy, which included a combination of dietary, exercise and behavioral strategies, delivered by a research dietician and trained research assistants, as described previously [27]. Within two weeks of randomisation, women attended a planning session with a research dietician. The information presented was subsequently reinforced during contact with the research dietician at 28 weeks’ gestation, and with trained research assistants (via telephone call at 22, 24, and 32 weeks’ gestation and a face-face visit at 36 weeks’ gestation).

Women randomised to receive Standard Care continued their pregnancy care according to local hospital guidelines, which did not include routine provision of dietary, lifestyle and behavioural advice related to diet or gestational weight gain.

The economic evaluation took the perspective of the health care system, and compared the costs directly associated with the additional use of resources from the time of randomisation until six weeks postpartum. Health outcomes for both the woman and her infant were considered in the clinical trial, but only those outcomes for which the intervention achieved a statistically significant improvement are included in the cost-effectiveness analysis, namely high infant birth weight and respiratory distress syndrome. Costs of outpatient occasions of service were calculated as the number of occasions each service was encountered, multiplied by the unit cost for that service. Table 1 presents the unit costs for the non-inpatient service encounters for which data were collected for all women randomised, including general practitioner or obstetrician/physician visits, midwifery visits, antenatal anaesthetist consultation, dietician visits, and antenatal emergency department attendance. Unit costs for these items were taken from hospital cost databases and the Medicare Benefits Schedule. Patient-level inpatient costs for the woman (including delivery), and neonatal care for the infant were available for each woman randomised to the trial utilising computerised inpatient cost information systems.

**Table 1:** **Unit costs of providing an antenatal lifestyle intervention for women who are overweight/obese**

**Item**	**Unit Cost (Australian dollars)**
Antenatal Clinic Visit (Doctor or Midwife)^*^	$217
Antenatal General Practitioner Visit^+^	$70.30
Antenatal Anaesthetic Visit^*^	$184
Antenatal Physician Visit^*^	$217
Diabetes Educator Visit^*^	$217
Provision Lifestyle Intervention^*^	$195

Service encounters collected from the trial data for all women randomised.

Unit costs were derived from the hospital cost databases (*) and the Medicare Benefits Schedule (+). Patient-level costings were available for all inpatient episodes.

Mean costs and effectives in the treatment groups allocated at the time of randomisation were compared, and incremental cost effectiveness ratios (ICERs) and confidence intervals (95%) calculated. Bootstrapping (using 1,000 resamples) was used to confirm the estimated confidence intervals, and to generate acceptability curves that represent the probability of the intervention being cost-effective at alternative monetary equivalent values for the main outcomes (avoiding high infant birth weight, and avoiding infant respiratory distress syndrome) [31].

Health related quality of life of the woman was assessed at baseline and at regular intervals to 4 months post-partum, by self-completion of the SF36-Health Survey Questionnaire [32]. The 36 items were combined into eight multi-item summary scores, and the Bayesian non-parametric conversion algorithm was used to generate health-related quality of life (utility) weights on a 0 (dead) to 1 (full health) scale [33]. Differences in utility profiles over the trial follow-up period were compared between the intervention and control group.

Consistent with the primary analysis of the LIMIT trial [27], economic analyses were adjusted for the stratification variables BMI category (25.0-29.9 kg/m^2^ vs ≥30.0 kg/m^2^), parity (parity 0 vs parity 1 or more), and centre of recruitment, and additionally for maternal age, and socio-economic status. All data were analysed according to the original treatment assignment using intention to treat principles.

## Results

Of the 2,212 women randomised to the LIMIT trial, 1,108 were randomised to the Lifestyle Advice Group, and 1,104 to the Standard Care Group. There were no statistically significant differences in the baseline characteristics of women randomised between the two treatment groups (Table 2) [27], or in health service encounters, with the exception of visits to the diabetes educator which were increased in women receiving Lifestyle Advice (Table 3). For clinical infant outcomes, as reported previously, infants born to women following lifestyle advice were significantly less likely to have birth weight above 4.0 kg (Lifestyle Advice 164/1075 (15.22%) versus Standard Care 201/1067 (18.79%); aRR 0.82; 95% CI 0.68 to 0.99; Number Needed to Treat (NNT) 28; 95% CI 15 to 263; p = 0.04) [27]. Furthermore, infants born to women following lifestyle advice were significantly less likely to have birth weight above 4 · 5 kg (2 · 15% versus 3 · 69%; aRR 0 · 59; 95% CI 0 · 36 to 0 · 98; p = 0 · 04), or respiratory distress syndrome (1 · 22% versus 2 · 57%; aRR 0 · 47; 95% CI 0 · 24 to 0 · 90; p = 0 · 02), particularly moderate or severe disease, and had a shorter length of postnatal hospital stay (3.94 ± 7.26 days versus 4.41 ± 9.87 days; adjusted ratio of means 0.89; 95% CI 0.82-0.97; p = 0.006) when compared with infants born to women who received standard care [30]. No significant differences in the health-related quality of life (utility) of women were identified between the two treatment groups over the follow-up period.

**Table 2:** **Baseline characteristics at trial entry [** 27]

***Characteristic***	***Lifestyle advice***	***Standard care***	***Total***
	(N = 1105**)	(N = 1097**)	(N = 2202**)
Maternal Age (Years)^*^	29.3 (5.4)	29.6 (5.6)	29.4 (5.5)
Gestational Age at Entry (Weeks)^+^	14.0 (11.9-17.0)	14.1 (11.9-17.0)	14.1 (11.9-17.0)
Body Mass Index (kg/m^2^)^+^	31.0 (28.1-35.9)	31.1 (27.7-35.6)	31.1 (27.9-35.8)
Body Mass Index Category^#^			
BMI 25.0-29.9	458 (41.4)	468 (42.7)	926 (42.1)
BMI 30.0-34.9	326 (29.5)	318 (29.0)	644 (29.2)
BMI 35.0-39.9	202 (18.3)	183 (16.7)	385 (17.5)
BMI > =40.0	119 (10.8)	128 (11.7)	247 (11.2)
Public Patient^#^	1081 (97.8)	1067 (97.3)	2148 (97.5)
Weight (kg)^*^	88.6 (17.3)	88 · 2 (17.6)	88.4 (17.4)
Height (cm)^*^	164.9 (6.6)	164 · 8 (6.5)	164.8 (6.6)
Race^#^			
Caucasian	995 (90.0)	998 (91.0)	1993 (90.5)
Asian	26 (2.4)	34 (3.1)	60 (2.7)
Indian	40 (3.6)	35 (3.2)	75 (3.4)
Other	44 (4.0)	30 (2.7)	74 (3.4)
Smoker^#^	154 (13.9)	126 (11.5)	280 (12.7)
Nulliparous^#^	457 (41.4)	441 (40.2)	898 (40.8)
Previous Preterm Birth^#^	57 (5.2)	59 (5.4)	116 (5.3)
Previous Pre-eclampsia^#^	46 (4.2)	51 (4.6)	97 (4.4)
Previous Stillbirth^#^	13 (1.2)	6 (0.5)	19 (0.9)
Previous Neonatal Death^#^	11 (1.0)	7 (0.6)	18 (0.8)
Previous Caesarean Section^#^	197 (17.8)	214 (19.5)	411 (18.7)
Family History of Diabetes^#^	288 (26.1)	290 (26.4)	578 (26.2)
Family History of Hypertension^#^	389 (35.2)	369 (33.6)	758 (34.4)
Family History of Heart Disease^#^	187 (16.9)	179 (16.3)	366 (16.6)
Index of Socio-economic Disadvantage^			
Unknown	2 (0.2)	1 (0.1)	3 (0.1)
Quintile 1 (Most Disadvantaged)	340 (30.8)	321 (29.3)	661 (30.0)
Quintile 2	271 (24.5)	264 (24.1)	535 (24.3)
Quintile 3	173 (15.7)	174 (15.9)	347 (15.8)
Quintile 4	150 (13.6)	178 (16.2)	328 (14.9)
Quintile 5 (Least Disadvantaged)	169 (15.3)	159 (14.5)	328 (14.9)

* = mean and standard deviation.

^+^ = median and interquartile range.

^#^ = number and %.

^= Socioeconomic index as measured by SEIFA.

** = Includes all women randomised who did not withdraw consent to use their data.

**Table 3:** **Health services utilisation from randomisation to 6 weeks post-partum**

**Service**	**Lifestyle advice**	**Standard care**	**Adjusted treatment effect (95% CI)**	**Adjusted P value**
	**N = 1,105**	**N = 1,097**		
**Antenatal Services**				
Antenatal Clinic Visit*	7.47 (±2.68)	7.38 (±2.65)	0.09 (−0.15 to 0.31)	0.46
General Practitioner Visit*	1.09 (±2.66)	1.12 (±2.74)	−0.05 (−0.28 to 0.18)	0.67
Antenatal Anaesthetic Visit*	0.21 (±0.45)	0.22 (±0.45)	−0.01 (−0.49 to 0.02)	0.49
Antenatal Physician Visit*	0.65 (±1.52)	0.57 (±1.44)	−0.08 (−0.05 to 0.20)	0.24
Diabetes Educator Visit*	0.38 (±1.40)	0.25 (±0.99)	−0.14 (−0.24 to 0.03)	0.01
**Birthing Services**				
Induction of Labour^+^	390 (36.25)	378 (35.39)	1.03 (0.92 to 1.15)	0.63
Caesarean birth^+^	370 (34.46)	389 (36.50)	0.95 (0.85 to 1.06)	0.34
**Neonatal Services**				
Admission to neonatal nursery^+^	394 (36.61)	385 (36.05%)	1.00 (0.90 to 1.12)	0.99
Need for respiratory support^+^	65 (6.09)	77 (7.20%)	0.84 (0.61 to 1.15)	0.27

* = Data presented as mean and standard deviation.

+ = Data presented as number and percentage.

Treatment effect represents adjusted difference in means (or relative risk ratio) with 95% confidence intervals.

Adjusted analyses included the stratification variables BMI category, parity and centre, in addition to adjustment for maternal age, and socioeconomic status.

### Health service use and direct outpatient- and inpatient costs for study participants

The mean cost associated with providing the Lifestyle Advice was $320.12 ± $130.97. Mean antenatal outpatient costs were $83.40 higher for women randomised to the Lifestyle Advice Group, compared with women receiving Standard Care (Lifestyle Advice Group $2116.39 ± $895.14 versus Standard Care $2032.99 ± $805.63; p = 0.022) (Table 4). However, mean costs were lower, albeit not reaching statistical significance, for women in the Lifestyle Advice Group, reflecting a mean of $281.01 savings in inpatient costs (Lifestyle Advice Group $6125.04 ± $4640.78 versus Standard Care Group $6404.05 ± $5274.54; p = 0.185), and $168.02 savings in infant hospitalisation costs (Lifestyle Advice Group $2699.64 ± $13068.48 versus Standard Care Group $2867.66 ± $12344.64; p = 0.756).

**Table 4:** **Cost of health services utilisation after randomisation for women and their infants**

**Service component**	**Lifestyle advice**	**Standard care**	**Adjusted treatment effect (95% CI)**	**Adjusted P value**
	**N = 1,105**	**N = 1,097**		
**Maternal Outpatient Services**				
Antenatal Clinic Visit	$1580.47 ($626.50)	$1558.16 ($625.02)	$22.30 (−$27.05 to $65.39)	0.416
(Obstetrician/Physician/Midwife)				
General Practitioner Visit	$118.97 ($204.49)	$115.42 ($210.07)	$3.55 (−$14.33 to $16.59)	0.689
Antenatal Anaesthetic Visit	$38.41 ($83.16)	$40.83 ($82.48)	-$2.43 (−$8.58 to $3.25)	0.477
Diabetes Educator Visit	$74.42 ($289.80)	$47.47 ($203.46)	$26.95 ($7.24 to $49.11)	0.008
Emergency Department Presentation	$305.06 ($466.84)	$272.18 ($380.69)	$32.88 (−$6.92 to $55.68)	0.127
***Total Outpatient Services***	***$2116.39 ($895.14)***	***$2032.99 ($805.63)***	***$83.40 ($38.25 to $140.50)***	***0.022***
***Provision of Antenatal Dietary & Lifestyle Advice***	***$320.12 ($130.97)***	N/A	N/A	N/A
***Maternal Inpatient Services***	***$6125.04 ($4640.78)***	***$6406.05 ($5274.54)***	***-$281.01 (−$289.31 to $448.38)***	***0.185***
***Neonatal Inpatient Services***	***$2699.64 ($13068.48)***	***$2867.66 ($12344.64)***	***-$168.02 (−$748.22 to $18.47)***	***0.756***
**Total Costs Incurred**	**$11261.19 ($14573.97)**	**$11306.70 ($14562.02)**	**-$45.51 (−$1349.26 to $1003.54)**	**0.094**

Data presented as mean and standard deviation.

Treatment effect represents adjusted difference in means with 95% confidence intervals.

Adjusted analyses included the stratification variables BMI category, parity and centre, in addition to adjustment for maternal age, and socioeconomic status.

Overall, therefore, the increase in mean costs associated with providing the intervention was offset by savings associated with improved immediate neonatal outcomes, rendering the intervention cost neutral (Lifestyle Advice Group $11261.19 ± $14573.97 versus Standard Care Group $11306.70 ± $14562.02; p = 0.094).

### Costs and consequences, and cost effectiveness of providing lifestyle advice

The unadjusted bootstrapped estimate of the difference in the proportion of infants with birth weight above 4 kg was 0.019 (95% CI 0.011 to 0.025), and infant respiratory distress syndrome was 0.0123 (95% CI 0.009 to 0.017). The incremental cost effectiveness ratio in preventing one additional infant with birth weight above 4 kg was $2395.26 (95% CI $2198 to $2411), and in preventing one additional infant with moderate to severe respiratory distress syndrome was $3700 (95% CI $3589 to $3755).

**Figure 1:**
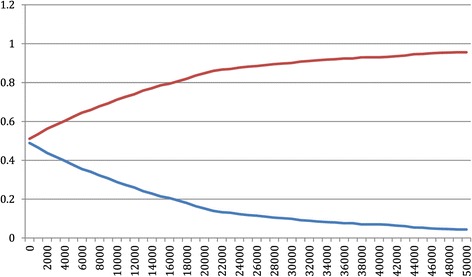
**Cost-effectiveness acceptability curve for infant birth weight below 4 kg.** Legend: Red line represents the cost effectiveness acceptability of achieving infant birth weight below 4 kg in the Standard Care Group; Blue line represents the cost effectiveness acceptability of achieving infant birth weight below 4 kg in the Lifestyle Advice Group.

**Figure 2:**
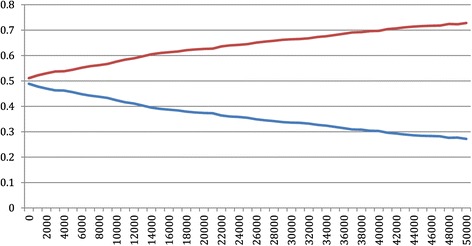
**Cost-effectiveness acceptability curve for preventing one infant developing moderate to severe respiratory distress syndrome.** Legend: Red line represents the cost effectiveness acceptability of preventing one infant developing moderate to severe respiratory distress syndrome in the Standard Care Group; Blue line represents the cost effectiveness acceptability of preventing one infant developing moderate to severe respiratory distress syndrome in the Lifestyle Advice Group.

Figures 1 and 2 represent the probability that providing an antenatal lifestyle intervention is cost-effective, in preventing both birth weight above 4 kg and moderate to severe respiratory distress syndrome, given alternative equivalent monetary values.

If a value of $20,000 is used as a threshold value for avoiding an additional infant with birth weight above 4 kg, the probability of the intervention being cost-effective is 0.85, compared with 0.15 in providing standard care (i.e. 1 minus the probability of cost-effectiveness for the intervention). This probability of intervention cost-effectiveness reaches almost 0.95 for the Lifestyle Advice Group if the maximum threshold willingness to pay increases from $20,000 to $45,000. Using the same threshold value of $20,000 for avoiding an additional infant with moderate or severe respiratory distress syndrome, the probability of cost-effectiveness is 0.64 in providing lifestyle advice. Additionally if we consider the maximum threshold value as $45,000, the probability of cost-effectiveness increases from 0.64 to nearly 0.73 for the Lifestyle Advice Group.

## Discussion

Our randomised trial is the largest reported to date evaluating the effect of an antenatal lifestyle intervention for women who are overweight or obese during pregnancy, and the first to report a robust economic evaluation of the associated costs and consequences. The findings of this health economic analysis conducted in parallel with a large-scale randomised trial indicate that the provision of an antenatal dietary and lifestyle intervention for pregnant women who are overweight or obese is not associated with statistically significantly increased costs or cost savings. While providing access to the intervention was associated with an increase in antenatal and dietician costs, this was offset by the savings associated with improved immediate infant birth outcomes and reduced hospitalisation costs. Using a monetary value of $20,000 as a threshold value for avoiding an additional infant with birth weight above 4 kg, the probability of cost-effectiveness of providing the antenatal intervention was 0.85, increasing to 0.95 with a monetary value of $45,000.

While the economic implications associated with overweight and obesity among non-pregnant individuals has been relatively well described [4,6-8], there is more limited information available relating to the health care costs associated with obesity during pregnancy. However, available literature indicates maternal overweight and obesity to be associated with increased costs of providing antenatal care, increased number of admissions and length of hospitalisation, and an overall increase in health care cost, when compared with women of normal BMI [20-24], reflecting in part an increase in the risk of obesity related maternal and infant complications [20]. Specifically, the mean total costs associated with pregnancy and postpartum care have been estimated to be 23% higher among women who are overweight, increasing to 37% higher among women who are obese, when compared with women of normal BMI [22], with suggestions that interventions costing less than £1171 per person could be cost effective in reducing healthcare utilisation costs among pregnant women who are obese [22]. The cost of providing dietary and lifestyle advice as described in the LIMIT randomised trial was approximately $320, or £179 per woman randomised, well below the above threshold.

The equally important and potentially more significant medium to longer-term implications of providing such a cost-neutral and effective antenatal intervention lies in the well-described association between high infant birth weight and subsequent risk of both childhood [34,35] and adulthood overweight and obesity [36,37], derived from several population-based cohort studies. Population cohorts have also identified consequent associations between high infant birth weight and subsequent cardiometabolic risk factors, including higher blood pressure, among children [38] which may persist into early adulthood [39]. Observational data from the United States involving 7,738 adolescents [40] highlights a significantly higher prevalence of obesity among children with birth weight above 4 kg. While children of high birth weight represented 12% of the cohort, this was disproportionately increased among children who were obese at age 14 years, where 36% of individuals had birth weight over 4 kg [40].

The economic implications associated with childhood obesity are considerable [41], with data from Australia identifying medical costs within the first five years of schooling to be $9.8 million greater for overweight or obese children at age 5 years, when compared with children of normal BMI [42]. Economic data from the United States indicate an increase in use of prescription medication, and both emergency and outpatient presentations among children who are overweight or obese, reflecting a cost of $14.1 billion annually [43], increasing by another $238 million per annum when accounting for increased inpatient admissions [44]. Conservative estimates suggest medical costs alone which are directly attributable to high childhood BMI are approximately $6.24 billion, with the loss of more than 2 million quality adjusted life years [41].

The medium to longer-term consequences of obesity in adulthood are substantial, with obesity considered the sixth most important factor contributing the global burden of disease [3], accounting for approximately 3.4 million adult deaths annually [6], through an increased risk of hypertension, cardiovascular disease, type-2 diabetes and malignancy [3]. The economic implications of treating complications related to adult obesity have variably been estimated at more than $58 billion, with $35.6 billion reflecting indirect costs [45]. Projections from the US highlight an increase in the proportion of total health-care expenditure to treat obesity and its complications [11], although as highlighted by others, direct international comparisons are exceedingly difficult [46].

There are limitations to our study. As reported previously [27], our trial population was predominantly of white Caucasian background and of high social disadvantage. Furthermore, 60% of eligible women declined to participate, reflecting both a lack of interest and time due to other commitments. The demographic characteristics of the women who participated in the LIMIT trial are similar to those of the broader population of women giving birth in South Australia [47], and therefore provide reassurance that our findings have wider clinical applicability. However, the transferability of the findings of our economic evaluation will likely vary with the similarities to both our trial population and health care system. While we used a standard assessment of social disadvantage in the SEIFA index, we were not able to assess in more detail the effects of occupation and household income. Furthermore, the analysis took the perspective of the health care institution, limiting our ability to evaluate household opportunity costs and financial implications from a societal perspective.

## Conclusions

Clearly, prevention, rather than treatment of obesity and obesity related complications should be the goal. Increasingly, there is recognition that the intra-uterine environment has a key role in the development of later health and disease [48], and therefore represents a critical period in the subsequent programming of obesity. Any antenatal intervention which successfully reduce the risk of high infant birth weight as was demonstrated in the LIMIT randomised trial [27], and particularly those which are cost effective as reported here, represent a public health strategy of significant potential in tackling the increasing problem of overweight and obesity, both in the short and longer-term [49,50]. Ongoing follow-up of participants into childhood will therefore be of great importance to determine the longer-term impact of the intervention on risk of obesity, and the associated economic implications.
